# Stable isotopic composition of Antarctic and Patagonian marine mammals collected before and during industrial-scale whaling: assessing the baseline for long-term changes in the marine ecosystem

**DOI:** 10.1098/rstb.2024.0227

**Published:** 2025-07-10

**Authors:** Evgeny A. Genelt-Yanovskiy, Anna S. Genelt-Yanovskaya, Maria Fontanals-Coll, Kweku Afrifa Yamoah, Oliver E. Craig, Richard C. Sabin, James D. Scourse

**Affiliations:** ^1^ Department of Earth and Environmental Sciences, University of Exeter, Penryn Campus TR10 9FE, UK; ^2^ BioArCh, Department of Archaeology, University of York, York YO10 5DD, UK; ^3^ Department of Zoology, The Natural History Museum, London SW7 5BD, UK

**Keywords:** Antarctic, bone collagen, museum collections, foraging ecology, stable isotopes, marine mammals

## Abstract

Great Antarctic expeditions, seal hunting and whaling industries left a legacy in natural history collections. To provide the basis for analysing the impact of whaling on marine ecosystem structuring, we conducted bulk isotope analysis from specimens of baleen whales (*Balaenoptera musculus* and *Balaenoptera physalus*) and seals (*Arctocephalus australis* and *Hydrurga leptonyx*) collected between 1843 and 1951 from the South Atlantic, Patagonian waters, Southern Ocean and Antarctic coastal seas, and preserved in the collection of Natural History Museum, London. Analysis of this material indicates the pre-whaling state of these environments and changes in the trophic position (TP) of whales and seals during the period of extensive human pressure. Being controlled for the Suess effect, *δ*^13^C values in *B. musculus*, *B. physalus* and *H. leptonyx* varied during the period of industrial-scale whaling. Bone collagen *δ*^15^N values and corresponding TP indicate possible trophic changes in *A. australis* and variability of the foraging areas of *B. musculus*. This study highlights the use of museum specimens for tracing historical trends associated with changes in the population structure and distribution of species, indicating long-term variability in their foraging ecology.

This article is part of the theme issue ‘Shifting seas: understanding deep-time human impacts on marine ecosystems’.

## 1. Introduction

The massive numbers of marine mammals and seabirds is among the most notable components of the Antarctic ecosystem. They play an important role as highly mobile consumers connecting the open sea pelagic and nearshore marine food webs. Antarctic and Subantarctic waters are crucial feeding grounds for many cetaceans, including baleen whales, attracted by seasonally high densities of krill [1,2]. Mainland Antarctica, Subantarctic islands and coasts of the Southern cool temperate province (Patagonia and Falkland Islands) houses vast breeding colonies of pinnipeds, and the productivity of the region is reflected in estimates that it contains more than a half of the global seal population.

Mammals function as keystone species, and serious depletion of their numbers can cause major changes in ecosystem functioning. Historically, overkill was the most important human activity affecting the abundance of marine mammals. Antarctic seas were effectively pristine prior to discovery of the continent in the eighteenth century. Whaling and sealing spread alongside the exploration of the region. Throughout the nineteenth and early twentieth centuries, the Antarctic was viewed as an almost limitless source of marine mammals to be hunted for skins, oil and other products. The development of seal hunting and subsequently whaling resulted in a severe decline in commercially important species. Seal hunting brought the Antarctic fur seal *Arctocephalus gazella* almost to extinction by the late 1700s to early 1800s in South Georgia, and the whaling industry peaked in the early 1900s when permanent whaling stations were opened at Grytviken (South Georgia) and Deception Island (South Shetland Islands) [3,4]. Following international moratoria on industrial whaling activities, several whale populations have been recovering over recent decades after a long period of decline, but recent census studies indicate that their abundance has still not attained pre-whaling numbers [5,6].

The significant decline in the abundance of marine mammals in the late nineteenth to early twentieth centuries caused by industrial-scale exploitation may well have had a significant impact on the marine ecosystem of Antarctica, as well as on the structure and distribution of populations of the species affected. Intensive whaling and seal hunting in Antarctica coincided with the period known as ‘Heroic Age of Antarctic exploration’, a series of naval and land expeditions between the late nineteenth century and 1920s. This period left a legacy in natural history museum collections around the world, including specimens of species targeted by industrial whaling and sealing. Museum specimens are increasingly important for gaining ecological and biological data and as carriers of geochemical and molecular information, important for studying rare, elusive and even extinct species. Historical museum ‘specimens of opportunity’ are often challenging to use owing to uncertainties in time and location of collection and in the quality of post-collection curation, but similar to subfossil specimens from excavations of archaeological or palaeontological sites, they can provide snapshots of past genetic diversity, spatial origin and diet [7–9]. Stable isotope analysis, most commonly the bulk *δ*^13^C and *δ*^15^N isotopes of marine species, can indicate latitudinal range shifts, migrations between nearshore and offshore environments, as well as changes in trophic structure [10].

Historical specimens of South Atlantic and Antarctic whales from various collections have become increasingly important in isotopic and genetic studies, being used to describe niche partitioning between fin (*Balaenoptera physalus*) and sei (*Balaenoptera borealis*) whales [11], changes in mitochondrial DNA diversity in blue (*Balaenoptera musculus*), humpback (*Megaptera novaeangliae*) and fin whales [12]. Isotopic analyses of collections representing the Antarctic fur seal (*A. gazella*) in Antarctica [13] and the South American fur seal (*Arctocephalus australis*) in Patagonia [14] indicate long-term latitudinal and longitudinal shifts in their prey distributions. The stable isotopic analysis approach is based on the relationship that carbon and nitrogen isotopes in the predator tissues reflect those of its prey in a predictable way [15]. Factors such as nutrient resources, composition of primary producers and regional geographical characteristics result in distinct isotopic patterns between different regions [16], allowing the use of stable isotope analysis for investigating large-scale movements of predators [16,17].

In this study, we measured *δ*^13^C and *δ*^15^N compositions of the oldest available Antarctic, Subantarctic and Patagonian marine mammal bone specimens of opportunity preserved in the Natural History Museum in London (NHMUK). These specimens represent the species most targeted by sealing and whaling activity and thus collected during the expeditions prior to, and during, the development of industrial scale harvesting. Following the timeline and southward trend of exploration of Subantarctic and Antarctic waters, the oldest specimens in the dataset correspond to the period of survey of the Strait of Magellan by the HMS *Alert* Expedition (1876−1884) and the James Clark Ross Expedition onboard HMS *Erebus* and HMS *Terror* (1838−1843). By focusing primarily on baleen whales—blue whale *B. musculus* and fin whale *B. physalus*, and two species of seals—leopard seal *Hydrurga leptonyx* and the South American fur seal *A. australis*—we explore the following research questions: (i) how have *δ*^13^C and *δ*^15^N stable isotopes varied during the onset of intensive whaling? (ii) can the observed variability in *δ*^13^C and *δ*^15^N be used for fingerprinting of foraging areas in highly mobile marine mammals? and (iii) can the observed trends in *δ*^15^N indicate spatial and temporal changes in the trophic position (TP) of the species?

## 2. Material and methods

### (a) Sampling

**Figure 1.**
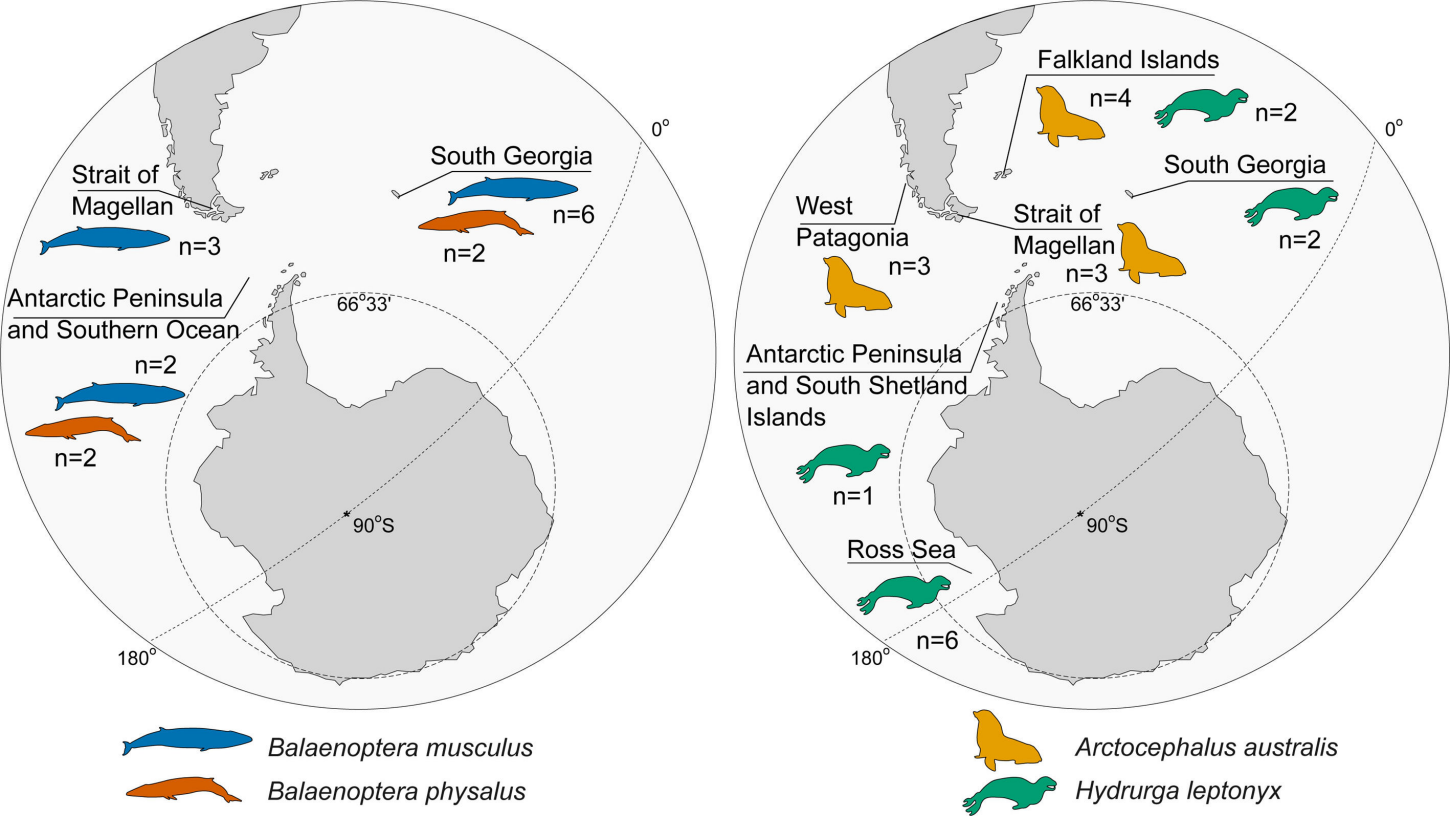
Geographical origin of specimens of marine mammals analysed in the study. Map redrawn from Google Earth. Detailed description of locations and NHMUK accession numbers are available in the electronic supplementary material S2, table 1 .

Samples were collected at the mammal collection of the NHMUK. The two key criteria for the selection of samples from the NHMUK collection were date of sampling and geographical origin identified either from the museum label directly or based on records related to the research cruise or programme. While the main focus of the study was on the South Georgia—Subantarctic islands biogeographical province according to, e.g. [18] and waters surrounding Antarctic Peninsula (Maritime Antarctic) (figure 1), whale and seal specimens from the Falkland Islands and Patagonia (Southern cool temperate province) were also considered, as these areas were visited during the Antarctic Expeditions and are, to a great extent, influenced by the wider Antarctic climate system [19,20]. In this study, we relied on species identification by the NHMUK, which included the DNA barcoding of all the fin and blue whale specimens implemented at the NHMUK facility. Before sampling, the surface of the target fragment of the bone was cleaned using a Dremel tool with the bristle polishing brush bit, and the cutting of bone fragment was implemented using handheld cutting tools and a Dremel with either the dental bur or carbide cutting disk. In total, 36 samples of bone fragments were analysed (table 1).

**Table 1.** Bone material and number of specimens used in the study.

species	*n*	years of collection	bone fragments sampled
*B. musculus*	11	1879–1951	periotic; ear bone; lumbar vertebra; shoulder blade; pelvic
*B. physalis*	4	1912–1951	periotic; ear bone; pelvic
*A. australis*	10	1876–1951	skull
*H. leptonyx*	11	1839–1963	skull

The initial weight of the bone samples was about 500 mg. Collagen was extracted and prepared for stable isotope analysis at the BioArCh laboratories, University of York (UK). The bone samples (approx. 200–300 mg) were demineralized using 0.4 M HCl at 4°C for several weeks, then rinsed with deionized water (milli-Q) and gelatinized with 0.001 M HCl at 70°C for 24–48 h. The supernatant containing the collagen was filtered using polyethylene Ezee filters (Elkay Laboratories, 9 ml, pore size 60−90 µm) and then 30 kDa Amicon Ultra-4 centrifugal filter units (Millipore, MA, USA). Samples were then frozen for 24−48 h at −20°C, lyophilized and weighed into tin capsules (0.4−0.6 mg per duplicate) for stable isotope analysis.

### (b) Bulk stable isotope analysis

The BioArCh laboratories at the University of York’s Department of Archaeology conducted duplicate measurements of stable carbon and nitrogen isotope ratios. Using a Sercon EA-GSL elemental analyser linked to a Sercon 20−22 continuous flow isotope ratio mass spectrometer (Sercon, Crewe, UK), the ratios of ^13^C : ^12^C and ^15^N : ^14^N were assessed relative to standards (Vienna Peedee Belemnite (V-PDB) for carbon and air for nitrogen) and reported in delta notation (*δ*) in parts per mil (‰). Stable carbon and nitrogen isotope ratios were measured using a Sercon HS2022 continuous flow isotope ratio mass spectrometer at BioArCh, University of York (UK).

Stable carbon and nitrogen isotope ratios were calibrated relative to VPDB and AIR scales using the standard reference materials IsoAnalytical Cane (*δ*^13^C = −11.64‰ ± 0.03), Sigma Methionine (*δ*^13^C = −35.83‰ ± 0.03, *δ*^15^N = −0.76‰ ± 0.05) and IAEA N2 (*δ*^15^N = 20.41‰ ± 0.12). Uncertainty was monitored using the standard reference materials Sigma fish gel (*δ*^13^C = −15.27‰ ± 0.04, *δ*^15^N = 15.21‰ ± 0.13), isoanalytical alanine (*δ*^13^C = −23.33‰ ± 0.10, *δ*^15^N = −5.56‰ ± 0.14) and isoanalytical soy (*δ*^13^C = −25.22‰ ± 0.03, δ^15^N = 0.99‰ ±0.07). Precision (u(Rw)) was determined to be ±0.165‰ for *δ*^13^C and ±0.124‰ for *δ*^15^N based on repeated measurements of calibration standards, check standards and sample replicates. According to calibration and check standards (table 1; electronic supplementary material, S1), measurement precision (ssrm, pooled measured s.d. of calibration and check standards) was ±0.151‰ for *δ*^13^C (d.f. = 28) and ±0.124‰ for *δ*^15^N (d.f. = 24). All the samples (42 out of 42) were analysed in duplicate (table 2; electronic supplementary material, S1). The measurement precision specific to samples (srep, pooled s.d. of sample replicates) was ± 0.094‰ for *δ*^13^C (d.f. = 47) and ±0.050‰ for *δ*^15^N (d.f. = 47). Accuracy (*u*(bias)) was determined to be ±0.091‰ for *δ*^13^C and ±0.159‰ for *δ*^15^N based on the difference between observed and reported *δ* values (RMSbias) and long-term standard deviations (*u*(Cref)) of check standards. Total analytical standard uncertainty (uc) was estimated to be ±0.188‰ for *δ*^13^C and ±0.204‰ for *δ*^15^N [22].

**Table 2.** Oceanic Suess effect correction values extracted from doi:10.1594/PANGAEA.872004 (see [21] for details). (*n*, number of point estimates of total accumulated Suess effect in the surface (0 metre) water layer. Total CSE, mean value of the total oceanic Suess effect for each region. The *a*-value, expected per-year *δ*^13^C decline (‰).)

	latitude range		longitude range		*n*	total CSE	*a*-value
Patagonia/Strait of Magellan	−49	−55	−77	−67	19	−0.91	−0.0065
Falkland Islands	−50.8	−52.5	−62	−57.4	2	−0.93	−0.0066
South Georgia	−53	−56	−38.7	−33.9	13	−0.73	−0.0052
South Shetlands	−61	−63	−63	−57	9	−0.75	−0.0054
Ross Sea	−71	−77	171	−160	173	−0.71	−0.0051

### (c) Data analysis

Basic descriptive statistics to explore variation for both *δ*^13^C and *δ*^15^N within the bone samples were performed using R [23] (with RStudio [24]). Raw *δ*^13^C values were normalized for potential lipid contamination. The resulting *δ*^13^C_normalized_ provides an estimate of *δ*^13^C that is normalized for the effects of lipid concentration on *δ*^13^C and is comparable to the *δ*^13^C after direct chemical lipid extraction [25].

Obtained *δ*^13^C_normalized_ values were then corrected for the oceanic Suess effect—the depletion in the *δ*^13^C isotopic composition of dissolved inorganic carbon (DIC) in oceans owing to the increase in anthropogenic CO_2_ released into the atmosphere since the Industrial Revolution [26–29]. Calculations were performed using the following equation introduced by Hilton *et al*. [26], modified by Misarti *et al*. [29] and later presented within the SuessR package [30]:

Suess effect correction factor = ***a*** × exp(***b*** × 0.027),

where ***a*** is a constant reflecting the maximum observed rate of *δ*^13^C decline in surface waters DIC in a specific region, ***b*** is the year of sample collection minus 1850 (taken as the onset of the Industrial Revolution) and 0.027 is the parameter value obtained by [26] after fitting an exponential curve to the global ocean *δ*^13^C data from 1945 to 1997 published by [31]. The values of total accumulation of Suess effect for the constant ***a*** were obtained from [21] for the surface waters (0 m depth layer in a database available under the doi:10.1594/PANGAEA.872004). These values were obtained and averaged for five regions (table 2). Per-year ratios of ***a*** were obtained by dividing these values by 140, i.e. the number of years between the year of 1850 (Industrial Revolution) and 1990 (start of the main decade of collecting data in [21]).

Bone *δ*^15^N signatures were converted to the TP using the following equations [32,33]:


$$\begin{array}{rl} TP & =2+(\delta^{15}N_{sample}–\delta^{15}N_{POM})/TEF,and\\ TP & =2+(\delta^{15}N_{sample}–\delta^{15}N_{POM}–TEF_{mmt})/TEF, \end{array}$$

where *δ*^15^N_sample_ is the nitrogen value in marine mammal bone, POM is the *δ*^15^N value of marine particulate organic matter, and TEF/TDF is the trophic enrichment (determination) factor (2–3.4) in *δ*^15^N for marine mammals [32,34–38]. TEF can be highly variable among species and foraging areas, and thus, it is difficult to estimate in large marine mammals who migrate long distances. We used two approaches for calculating TEF for baleen whales following recent publications [35]. The first uses TEF values previously obtained for the fin whale [34], later called as ‘Borrell TEF’. The second is typically accepted and widely used for marine ecosystem TEF values from [38], hereafter ‘post TEF’. In the equation above, TEF_mmt_ is the tissue-specific trophic enrichment factor in *δ*^15^N; for baleen whale bone protein samples, TEF_mmt_ = 2.03 ± 0.71 was used [39].

Since the isotopic signatures of POM vary spatially and temporally in the study region [40], observed ranges in POM isotopic signature corresponding to the location of the specimen find site were used. Data on POM were obtained from previously published modern datasets; *δ*^15^N_POM_ = 0.6 for the Ross Sea, *δ*^15^N_POM_ = 1.7 for the South Shetlands, *δ*^15^N_POM_ = 1.5 for the South Atlantic sector of the Southern Ocean, *δ*^15^N_POM_ = 3.6 for South Georgia (range 2–5.4), *δ*^15^N_POM_ = 8.1 for the Strait of Magellan (range 5.6–12.2), *δ*^15^N_POM_ = 9.4 for the Falkland Islands, *δ*^15^N_POM_ = 9.4 for the Patagonian West Coast [40–45].

Consumer and source isotope data were analysed using the MixSIAR package in R 4.4.3 [23,46,47], where source data were composed of mean and s.d. from potential prey sources. Mixing models were applied to the datasets of the blue whale and South American fur seal, both demonstrating high variability in isotopic signatures. To build the potential regional dietary database for the blue whale (table 3), stable isotopic signatures of dominant krill species were obtained for five regions, from Antarctic shelf waters to Patagonian shelf waters [48,50–53]. Isotopic data on potential prey objects of the South American fur seal (table 3) were obtained based on recent studies of behaviour and dietary preferences of the species, and isotopic signatures were then mined from the literature [51,54,55]. All the used *δ*^13^C values of potential prey items passed the same Suess-correction procedures as the marine mammal data, and the difference between non-corrected and corrected values for the dietary source data varied between 0.29‰ and 0.38‰.

**Table 3.** Stable isotope signatures of potential prey items for the blue whale and South American fur seal, mined from the literature (see text for references) and used for the MixSIAR modelling.

prey group/species	region	*n*	*δ*^13^C		*δ*^15^N		source
			mean	s.d.	mean	s.d.	
*Euphasia superba*	South Shetland Islands	24	−26.87	0.94	3.63	0.38	Schmidt *et al*. [48]
*Euphasia superba*	South Georgia	26	−23.87	1.22	4.27	0.54	Schmidt *et al*. [48]
*Thysanoessa* spp*./* *Euphasia frigida*	polar front and southwest Patagonian shelf waters	7	−23.25	1.05	5.10	0.20	Schmidt *et al*. [48]
*Euphasia superba*	Antarctic shelf waters	43	−31.20	0.10	2.85	0.75	Schmidt *et al*. [48]
*Euphasia mucronata (Humboldt krill*)	west Patagonia shelf (Chile)	30	−16.04		12.37		Hückstädt *et al*. [49]
*Euphasia lucens*	east Patagonian shelf (Falkland Islands	3	−20.61	0.39	8.04	1.22	Büring *et al*. [50]
*Euphasia lucens*	Subantarctic (southeast Patagonian shelf waters)	7	−19.80	0.70	7.33	0.80	Ciancio *et al*. [51]
squid (*Doryteuthis gahi*)		10	−19.00	0.60	13.60	0.70	Ciancio *et al*. [51]
demersal fish (*Patagonotothen* spp.)		5	−19.30	0.60	14.80	1.00	Ciancio *et al*. [51]
small planktonic fish (*Sprattus fuegensis*)		25	−20.20	1.00	13.10	0.90	Ciancio *et al*. [51]
piscivorous fish (hake)		6	−14.90	0.30	17.20	0.40	Ciancio *et al*. [51]

Models were run with uninformative Bayesian priors on source proportion contributions and no random effects. The models ran three Markov chain Monte Carlo chains with 100 000 iterations, a burn-in of 50 000, and were thinned by 50. MixSIAR reports results as mean, s.d. and credible intervals for each posterior density distribution per prey source. Model convergence was checked using the diagnostic tests and plots available in MixSIAR [46,56,57].

To identify the within-group variability in dietary sources, SIMPER analysis was implemented in PAST v5.0 [58] using the matrix of obtained mean values of modelled proportion of diet. PAST v5.0 was also used to run PERMANOVA analysis applied to various among-group comparisons.

## 3. Results

### (a) Stable isotope data

**Figure 2.**
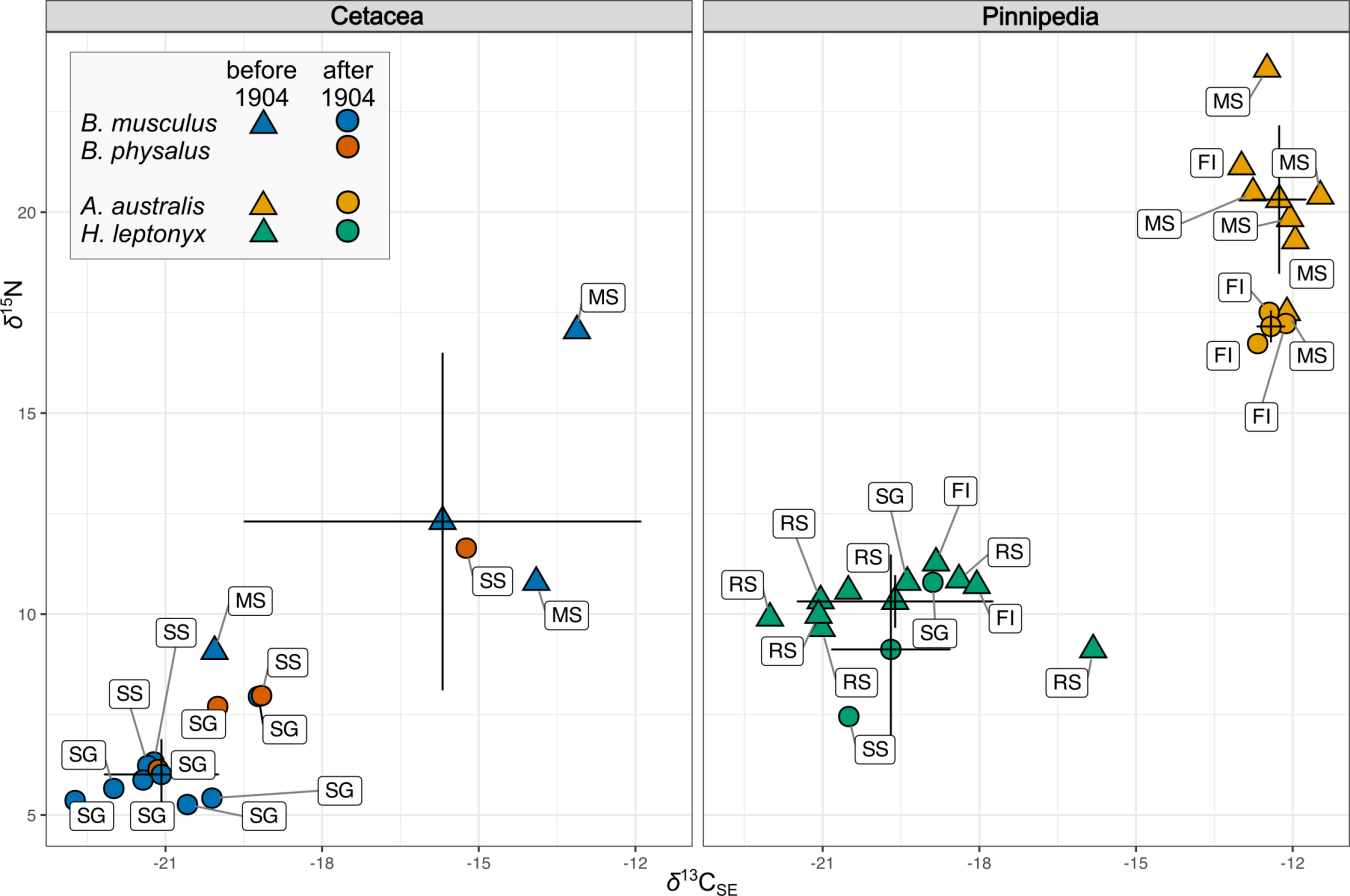
Suess corrected *δ*^13^C and *δ*^15^N biplot summarizing within-species variability of samples collected before (triangles) and after (circles) the opening of the whaling station at South Georgia in 1904. Points with error bars denote mean ± s.d. values for each period. Region codes: MS, Strait of Magellan and Patagonian West Coast; FI, Falkland Islands; SG, South Georgia; SS, Southern Ocean and South Shetland Islands; RS, Ross Sea.

The carbon-to-nitrogen (C : N) atomic ratio range from 3.29 to 4.3 in the analysed marine mammal specimens. Several specimens demonstrated signatures of lipid contamination based on the C : N mass values (electronic supplementary material, S2, table S1). Thus, a mathematical correction (see §2 for details) was applied to raw *δ*^13^C values for all specimens before subsequent analyses. The *δ*^13^C_normalized_ and *δ*^15^N values vary significantly among both blue whale and fin whale historical specimens studied (figure 2; table 4).

**Table 4.** Summary of stable isotope values (mean ± s.d.) of bone collagen samples of marine mammals collected between the 1880s and 1960s.

species	*n*	raw *δ*^13^C ± s.d.	*δ*^13^C_normalized_ ± s.d.	*δ*^13^C_SE_ ± s.d.	*δ*^15^N ± s.d.
*B. musculus,* blue whale	11	−19.86 ± 3.32	−19.64 ± 3.18	−19.61 ± 3.17	7.73 ± 3.57
*B. physalus,* fin whale	4	−19.10 ± 2.64	−18.91 ± 2.56	−18.88 ± 2.26	8.36 ± 2.33
*A. australis,* South American fur seal	10	−12.31 ± 0.48	−12.33 ± 0.45	−12.31 ± 0.45	19.37 ± 1.25
*H. leptonyx,* leopard seal	11	−20.09 ± 1.25	−20.00 ± 1.30	−19.97 ± 1.31	10.21 ± 1.04

The *δ*^13^C_normalized_ values of blue whale specimens vary both spatially and temporally between Patagonia and South Georgia with the range observed between −22.76‰ and −13.14‰, and similarly *δ*^15^N values vary between 5.26‰ and 17.05‰ (see the electronic supplementary material, S2, table S1 for details). In the fin whale, *δ*^13^C_normalized_ values range between −21.17‰ and −15.27‰, and *δ*^15^N values range between 6.13‰ and 11.64‰. The *δ*^13^C_normalized_ values in South American fur seals demonstrate a narrow range between −12.99‰ and −11.49‰, while *δ*^15^N values vary between 16.73‰ and 23.56‰. In leopard seals, *δ*^13^C_normalized_ values showed more pronounced variation (−22.01‰ to −15.83‰), compared to variation in *δ*^15^N between 7.45‰ and 11.27‰.

The Suess-corrected *δ*^13^C values demonstrate the same trends and do not deviate more than 0.11‰ from *δ*^13^C_normalized_ values, and the mean value for all species is 0.027‰ (figure 2; table 4). The maximum correction value of the raw *δ*^13^C to take into account the potential effects of lipid contamination, and oceanic Suess effect (0.97‰) was applied to the blue whale specimen from South Georgia collected in 1914 (ZC.1953.12.1.16). The sample from this specimen is characterized by a very high C : N mass ratio (4.3); thus, the raw *δ*^13^C value 23.7‰ has been corrected to −22.73‰. The complete set of individual *δ*^13^C and *δ*^15^N values, spatial variation of indices and detailed information about the origin of each specimen analysed in this study is presented in the electronic supplementary material, S2, table S1.

### (b) Pre- and post-1904 stable isotopic values

In total, 19 specimens predating the onset of industrial whaling in Antarctic waters were analysed, including three specimens of blue whale from Patagonian waters, seven specimens of the South American fur seal from the Falkland Islands and nine specimens of leopard seal (table 5; figure 2). The mean (±s.d.) Suess corrected *δ*^13^C_SE_ value over this period in blue whale samples is −15.69 ± 3.8, and mean *δ*^15^N = 12.3 ± 4.2, in the South American fur seal samples mean *δ*^13^C_SE_ = −12.26 ± 0.52 and mean *δ*^15^N = 20.32 ± 1.84, and leopard seal mean *δ*^13^C_SE_ = −20.04 ± 1.40 and mean *δ*^15^N = 10.39 ± 0.65 (table 5).

**Table 5.** Stable isotope ratios in bone collagen of NHMUK specimens predating the opening of the whaling stations at south Georgia and Deception Island in the early 1900s. (See the electronic supplementary material, S2, table S1 for the isotopic values of post-1904 samples in the dataset.)

NHMUK ID	species	location	year^a^	*δ*^13^C_normalized_	*δ*^13^C_SE_	*δ*^15^N
ZD.1880.7.28.19	*B. musculus*	Strait of Magellan**^a^**	March 1879	−20.07	−20.06	9.07
ZD.1879.8.21.9	*B. musculus*	Strait of Magellan	March 1879	−13.91	−13.90	10.79
ZD.1879.8.21.8	*B. musculus*	Strait of Magellan	February 1879	−13.14	−13.12	17.05
ZD.1852.3.11.5	*A. australis*	Falkland Islands	before 1850	−12.99	−12.99	21.13
ZD.1879.8.21.5	*A. australis*	Strait of Magellan	1878–1880	−12.12	−12.11	17.50
ZD.1880.7.28.12	*A. australis*	Strait of Magellan	1878–1880	−11.97	−11.95	19.29
ZD.1880.7.28.13	*A. australis*	Strait of Magellan	1878–1880	−11.49	−11.47	20.40
ZD.1880.7.28.14	*A. australis*	Patagonian west coast	1878–1880	−12.51	−12.49	23.56
ZE.1950.11.14.3	*A. australis*	Patagonian west coast	04/01/1876	−12.06	−12.05	19.84
ZE.1950.11.14.4	*A.australis*	Patagonian west coast	04/01/1876	−12.78	−12.76	20.48
ZD.1843.1.8.4	*H. leptonyx*	Antarctic seas	1843	−22.01	−22.01	9.90
ZD.1846.4.15.23	*H. leptonyx*	Antarctic seas	1843	−18.39	−18.39	10.85
ZD.1846.4.15.24	*H. leptonyx*	Antarctic seas	1843	−21.05	−21.05	10.33
ZD.1880.7.28.5	*H. leptonyx*	Falkland Islands	1878–1880	−18.84	−18.83	11.27
ZD.1885.10.20.1	*H. leptonyx*	Falkland Islands	before 1885	−18.07	−18.05	10.71
ZD.1893.9.14.1	*H. leptonyx*	South Georgia	before 1893	−19.39	−19.38	10.79
ZD.1908.2.20.54	*H. leptonyx*	Ross Sea	1901–1904	−21.04	−21.02	9.64
ZD.1908.2.20.56	*H. leptonyx*	Ross Sea	1901–1904	−20.53	−20.51	10.57
GERM.325.e	*H. leptonyx*	Antarctic seas	1839–1843	−21.09	−21.09	9.97

^a^ Strait of Magellan dataset consists of material from the Strait itself and surrounding archipelagoes along the Chilean Pacific coastline/west coast of Patagonia (see the electronic supplementary material, table S1, S2 for details).

Among the post-1904 specimens (figure 2), the *δ*^13^C_SE_ mean value for the blue whale is −21.08 ± 1.1, and the mean *δ*^15^N value 6.01 ± 0.88 (*n* = 8). In South American fur seals, the mean *δ*^13^C_SE_ value is −12.26 ± 0.52 and *δ*^15^N mean value 17.16 ± 0.40 (*n* = 3). In leopard seals, the mean *δ*^13^C_SE_ value is 19.70 ± 1.14 and *δ*^15^N mean value 9.29 ± 1.70 (*n* = 2).

Differences were most pronounced in the *δ*^15^N values for the South American fur seal (PERMANOVA *F* = 14.61, *p* = 0.041; figure 2), yet no difference in *δ*^13^C_SE_ values for this species has been found between the samples collected at the end of the nineteenth and in early twentieth centuries. Despite the large variability in *δ*^13^C_SE_ values in general, no significant changes in either *δ*^13^C_SE_ or *δ*^15^N in leopard seals have been found. Changes in stable isotope signatures after 1904 in the blue whale are significant for both carbon and nitrogen with more pronounced changes in *δ*^15^N (PERMANOVA *F* = 38.94, *p* = 0.0011); differences in *δ*^13^C_SE_ are also significant (PERMANOVA *F* = 15.02, *p* = 0.0134).

**Figure 3.**
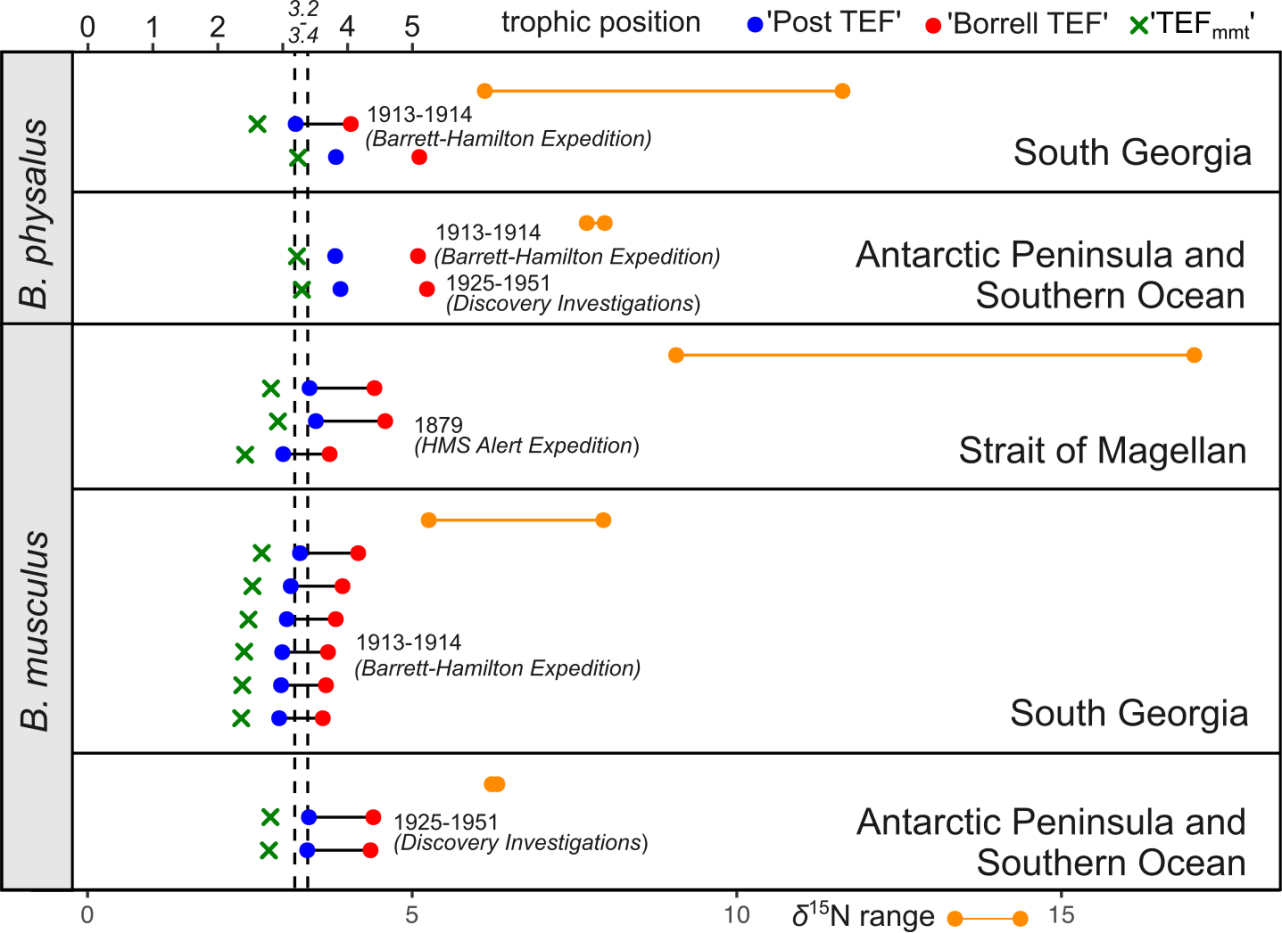
Trophic position (TP) estimates of individual baleen whale specimens and corresponding bone collagen *δ*^15^N ranges in each studied region. Blue dots, red dots and green crosses represent three TP calculation approaches based on different trophic enrichment factor (TEF) values (see §2 for details). Non-connected blue and red dots indicate putative geographical outliers. Line-connected orange dots mark the observed range of *δ*^15^N signatures in each region. Dashed lines represent expected range of TP for modern blue and fin whales (3.2−3.4) [59].

### (c) Estimation of trophic positions

The calculated TP varies between 3.2 and 5.2 in the fin whale and between 2.9 and 4.5 in the blue whale; averaged TP ± s.d. is 3.6 ± 0.51 in the blue whale and 4.3 ± 0.75 in the fin whale (figure 3), indicating several outlier values being higher or lower than the expected TP for these species. All the TP values were calculated based on the POM *δ*^15^N values specific to each region (see §2 for details). TP values calculated based on TEF_mmt_, i.e. corrected based on tissue-specific TEF, yield 2.4−2.9 (2.6 ± 0.21) in the blue whale, and 2.6−3.3 (3.1 ± 0.32) in fin whales. Considering the high migratory abilities of the studied species and that the origin of each specimen was indicated at NHMUK based on the locality of landing, observed higher outlier TP values can putatively indicate more northerly/temperate foraging areas and vice versa—outliers with TP lower than expected values can indicate more southern (closer to Antarctica) foraging areas.

Comparison of TP in different time periods and regions of sampling using PERMANOVA analysis indicates significant TP differences in the blue whale between the material from 1913 to 1914 (South Georgia) and 1925 to 1951 (Antarctic Peninsula and Southern Ocean) (PERMANOVA *F* = 2.96, *p* = 0.0354). Owing to high within-sample variability, no differences between the 1879 samples and the subsequently collected material were revealed using PERMANOVA. No differences were also found between fin whale specimens collected in two different periods (PERMANOVA *F* = 1.175, *p* = 0.663).

**Figure 4.**
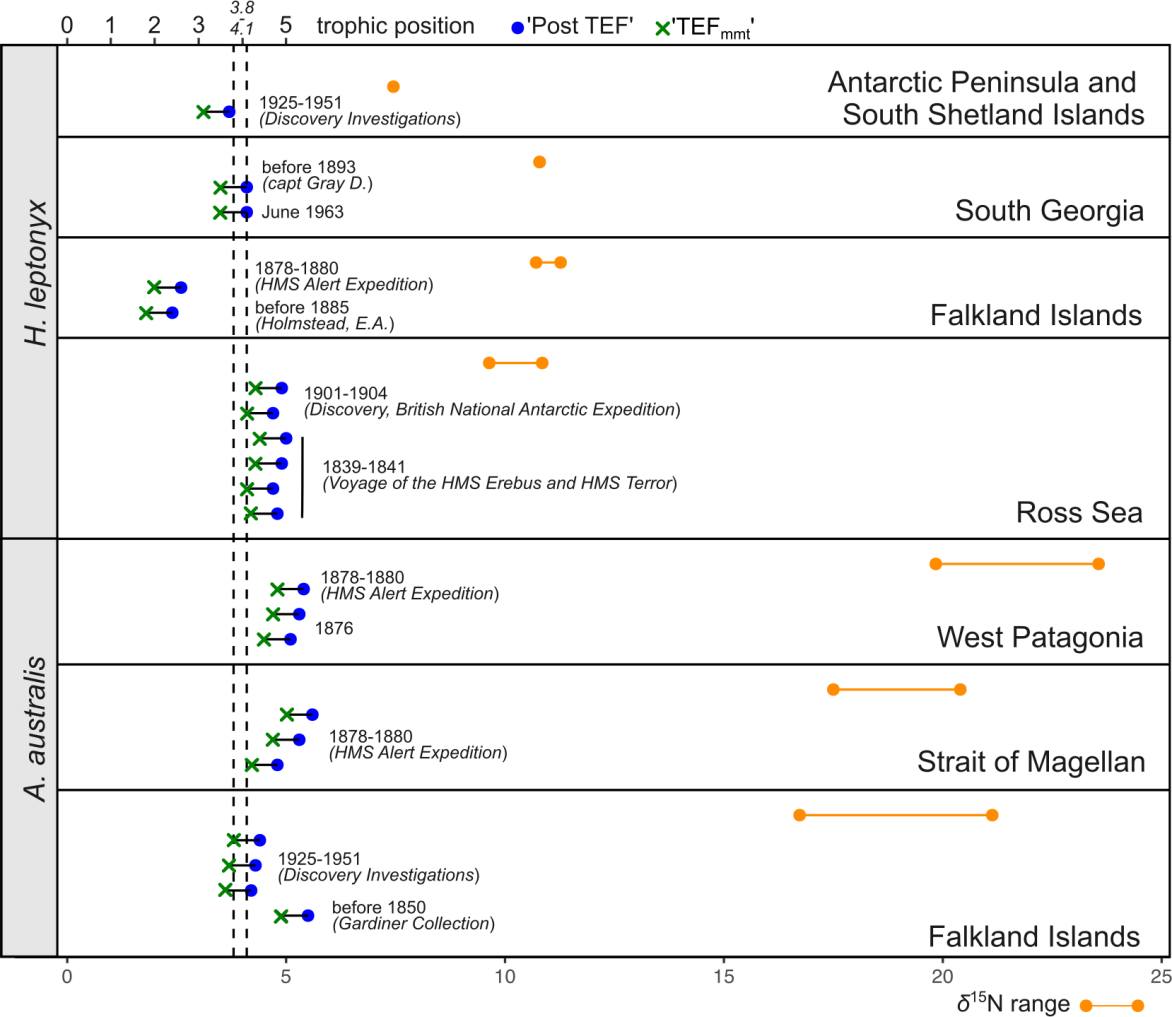
Trophic position (TP) estimates of individual leopard seal and South American fur seal specimens and corresponding bone collagen *δ*^15^N ranges in each studied region. Blue dots and green crosses represent three TP calculation approaches based on different TEF values (see §2 for details). Line-connected orange dots mark the observed range of *δ*^15^N signatures in each region. Dashed lines represent expected range of TP for modern seals (3.8−4.1) [59].

South American fur seal TP calculated using ‘post TEF’ varies from 4.2 to 5.6 with higher values in the Straits of Magellan and lower values in the Falkland Islands, with a mean value of 5 ± 0.53 (figure 4). Leopard seal TP varies between 2.4 and 5.0 (4.2 ± 0.93). The highest TP values in leopard seals are found in the Ross Sea, with lowest values in specimens from the Falklands. TP values calculated based on TEF_mmt_, vary between 3.6 and 5 (4.4 ± 0.53) in the South American fur seals, and between 2 and 4.4 (3.6 ± 0.93) in leopard seals.

South American fur seal specimens collected between 1850 and 1880s in the Falkland Islands, Strait of Magellan and west Patagonia do not show significant differences in TP (PERMANOVA *F* = 0.32, *p* = 76.84). The TP of this group of specimens collected before the 1880s significantly deviates from the TP of fur seals collected between 1925 and 1951 (Discovery Investigations) in the Falkland Islands (PERMANOVA *F* = 39.85, *p* = 0.0085). In the leopard seals, no temporal differences between the material collected in the 1830s and 1904 in the Ross Sea have been found (PERMANOVA *F* = 0.19, *p* = 0.6641). Comparison of all the material collected in the nineteenth century indicates a difference in specimens from the Falklands from the other material, indicating significantly lower TP compared to other localities (PERMANOVA *F* = 311.1, *p* = 0.0045).

### (d) Source prediction and foraging areas fingerprinting by MixSIAR

**Figure 5.**
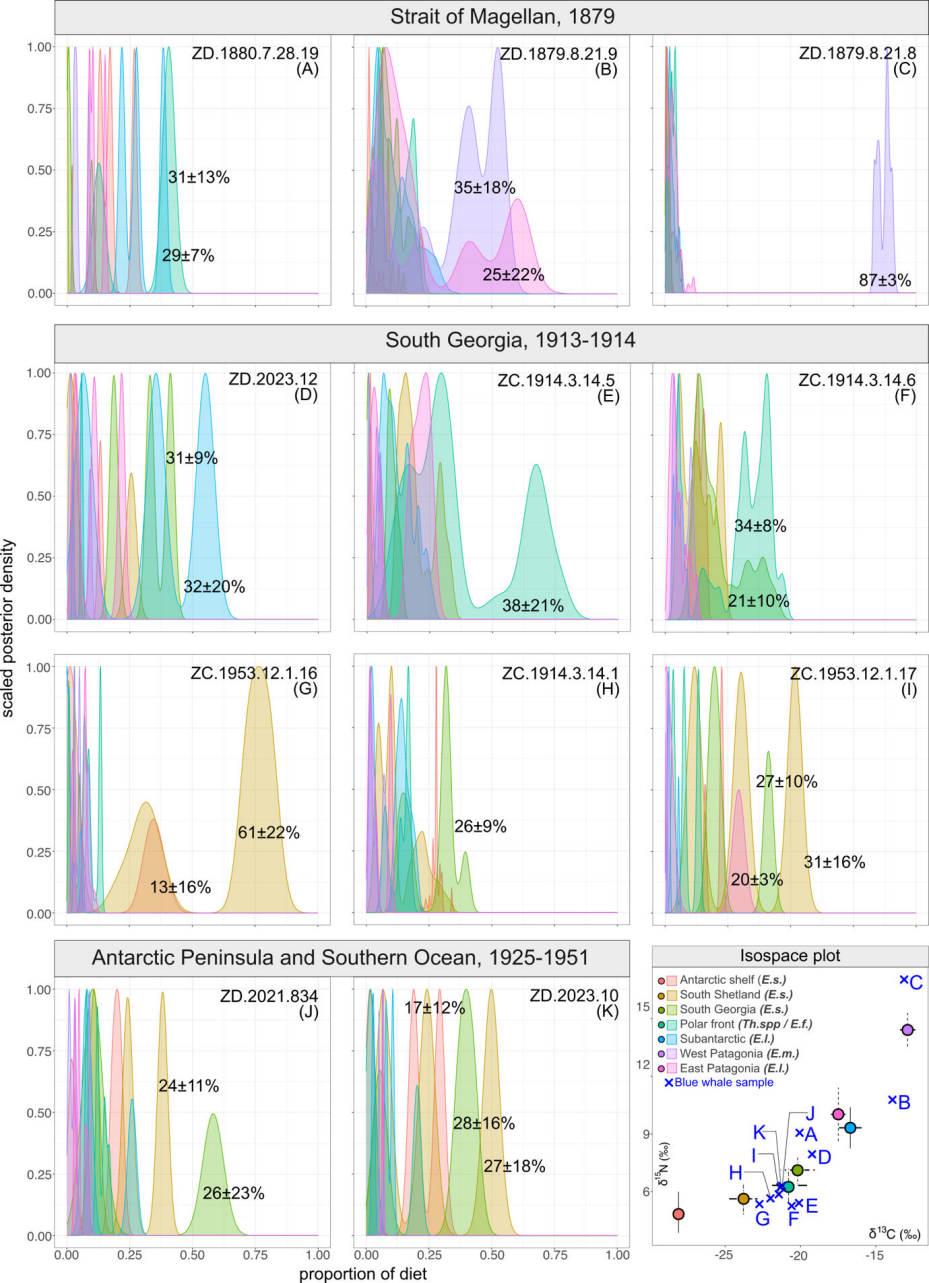
MixSIAR-estimated dietary source contributions (as Bayesian credibility intervals and posterior densities) for each studied specimen of the blue whale *B. musculus* from Strait of Magellan (A-K), South Georgia (D-I), and Antarctic Peninsula and Southern Ocean (J-K). Abbreviations indicate potential prey items: *E.s., Euphasia superba; Th.* spp,*—Thysanoessa* spp*.; E.f., Euphasia frigida; E.l., Euphasia lucens; E.m., Euphasia mucronata* (see §2 for details).

The Bayesian mixing modelling approach was applied to the datasets on the blue whale and South American fur seal, because these species demonstrate significant changes in TP over the period between the 1850s and 1950s. For the blue whale specimens, mixing models were used to evaluate the contribution of various regions as key foraging areas (figure 5). According to the MixSIAR reconstructions based on stable isotope data of five species of krill from seven geographical regions, most of the studied blue whale individuals had mixed dietary sources. The overall average dissimilarity statistics of SIMPER analysis, applied to the matrix of proportion of diet from mixing models, was 48% for the complete dataset of 11 whale specimens, with the highest contribution to the variability derived from krill from west Patagonia and South Shetland, respectively (*ca* 20% each).

Among the oldest specimens in our dataset, all collected in the Strait of Magellan in the 1870s, one specimen (figure 5C) demonstrates clear geographical affinity with the major contribution of krill from Chilean waters. For specimen ZD.1871.8.21.9 (figure 5B), the model suggests mixed sources originating from both east and west Patagonia (i.e. South Pacific and South Atlantic). By contrast, the MixSIAR model is not able to distinguish the single major foraging area for ZD.1880.7.28.19 (figure 5A). For the largest group of specimens in the dataset with similar geographical origin and time of collection, South Georgia (figure 5D–I), the SIMPER overall similarity score is 41%. MixSIAR is able to predict the dominant foraging area for only one specimen of this subset (figure 5G), the waters offshore the South Shetland Islands (61 ± 22% diet contribution). For the rest of the specimens, a relatively high contribution to the diet also contained krill from the polar front, the waters surrounding South Georgia and in the Subantarctic.

**Figure 6.**
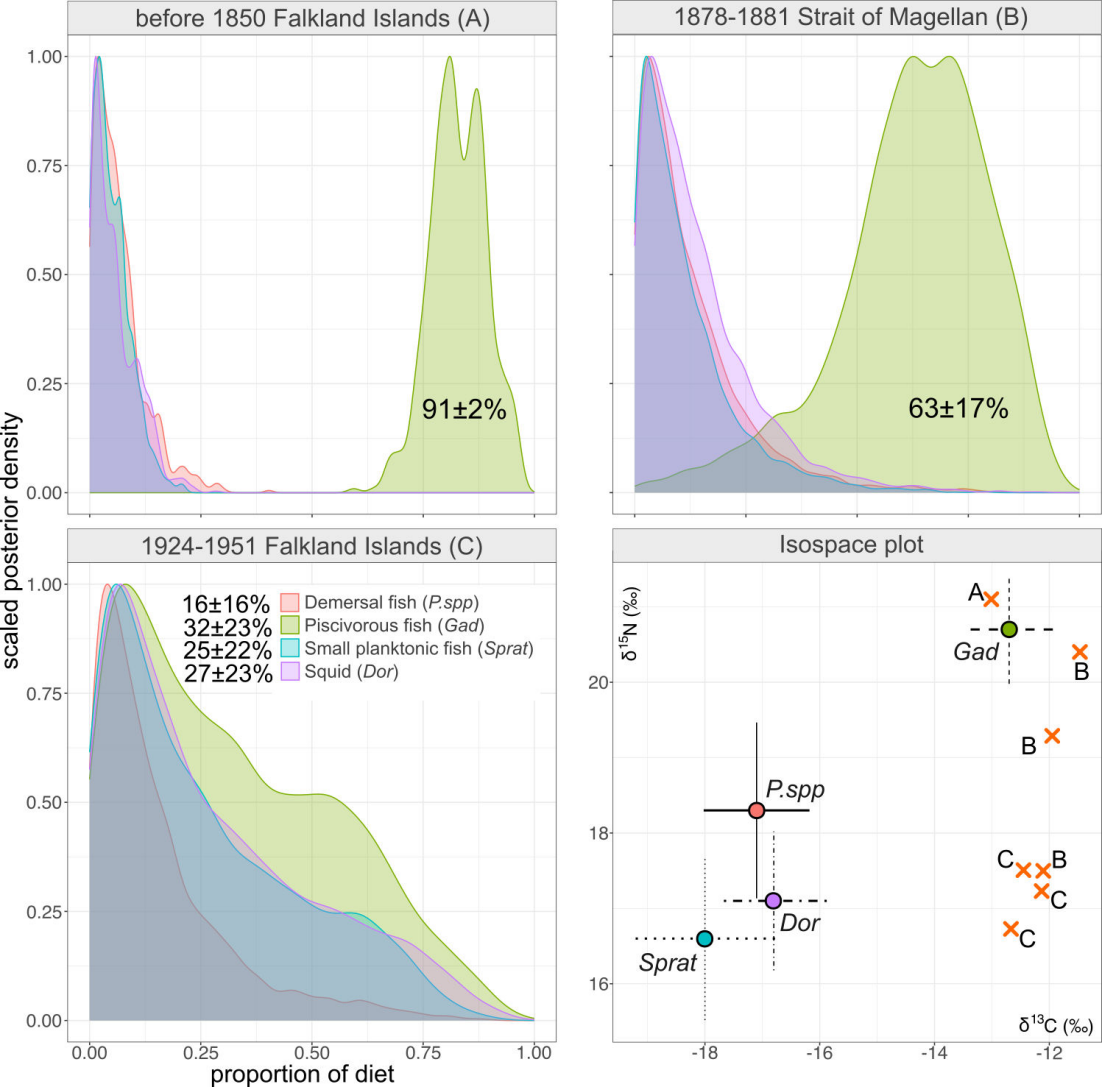
MixSIAR-estimated dietary source contributions (as Bayesian credibility intervals and posterior densities) for the South American fur seal *A. australis* Falkland Islands collected before 1850 (A), Strait of Magellan between 1878 and 1881 (B), and Falkland Islands between 1924 and 1951 (C). from*. P.* spp*., Patagonotothen* spp*.; Gad, Gadidae; Sprat, Sprattus fuegensis; Dor, Doryteuthis (Loligo) gahi.*

The MixSIAR model for the South American fur seals was applied to identify temporary changes in dietary sources of specimens from the Falkland Islands and Strait of Magellan. According to the mixing models, the highest contribution to the diet of the specimens from both regions in the nineteenth century was piscivorous fish species (figure 6A,B). On the contrary, the MixSIAR model was not able to distinguish any taxa with a major contribution to the diet for specimens dated between 1924 and 1951 from the Falkland Islands.

## 4. Discussion

The number of studies examining the impacts of industrial whaling during the twentieth century is increasing [5,11,12,45,60]. It is a priority to accumulate as much as possible data representing the baseline state of their populations prior to the onset of industrial-scale whaling. Despite the several uncertainties in the dataset, particularly the origin and often the unclear state of preservation of several specimens, this study provides a snapshot of stable isotope signatures of common Southern Hemisphere marine mammals during the period of extensive exploration of Antarctic waters in the nineteenth century and at the turn of the twentieth century.

Museum specimens represent a unique source of information about past populations and rare species [10], but in some cases it can be difficult to handle if their provenance is unclear [61]. For most of the specimens used in this study, we were able to detect the geographical origin and time of collection (electronic supplementary material, S2, table S1). The highest level of such uncertainty is linked to the material from the Discovery Investigations programme (1925−1951), including several specimens of blue whale*,* fin whale and South American fur seal. All the samples used from the Discovery Investigations were represented as belonging to this wide time interval. Several specimens in our dataset demonstrated deviations from the commonly accepted range of C : N atomic ratio between 2.9 and 3.3 [62]. Yet, since the isotopic signatures of these specimens did not deviate strongly from the other specimens from same locations and periods (electronic supplementary material, S2, table S1), they were retained in our dataset; a mathematical correction for potential lipid contamination was applied to all specimens [25].

Previously, correction of *δ*^13^C values for the oceanic Suess effect proved important for the analysis of long-term changes of isotopic signatures of consumers in the Southern Hemisphere. The oceanic Suess effect is generally considered as negligible for the Southern Ocean, and thus the Suess-correction of *δ*^13^C often is avoided in Antarctic studies [63]. A study of four species of penguins from the French Southern Ocean and Antarctic territories [64] demonstrated that Suess-corrected *δ*^13^C values did not change over time in Antarctic species from Adelie Land, but decreased in the Subantarctic and subtropical species. However, Suess-corrected *δ*^13^C values of Weddell seals from the Ross Sea decreased during the twentieth century [49], indicating changes in the primary productivity of this sector of the Antarctic. The strength of the oceanic Suess effect increases in lower latitudes north of the Subantarctic Front [21]. Since our dataset included specimens from the Subantarctic islands, Falkland Islands and Patagonia, where the Suess effect has been evaluated to be higher [65], and we used modern isotopic signatures of POM and potential prey items from various latitudes in trophic reconstructions, the correction was applied to all raw bone collagen *δ*^13^C values and all used modern datasets. The correction values did not alter the results based on raw *δ*^13^C values but were necessary for comparison of the carbon signatures among specimens from the same species collected at various locations during different periods.

We find pronounced variation in bone collagen *δ*^13^C and *δ*^15^N values across the studied museum specimens of two species of baleen whales (*B. musculus* and *B. physalus*) and in leopard seal (*H. leptonyx*). In the South American fur seal (*A. australis*), we observe temporal changes in *δ*^15^N and no variation in *δ*^13^C values. The observed intraspecific variation in isotopic signatures mostly represent the geographical origin of various specimens in our dataset but may also potentially indicate temporal variation in foraging strategies and in the distribution of highly mobile species. This can also be owing to the differences in sex and age of individuals, the parameters not always well referred to in museum records. Below we discuss the potential sources of the observed variation for each species.

Here, we use Bayesian mixing models as a fingerprinting tool to detect the potential foraging areas, as well as affinity to various geographical populations and subspecies of blue whale in the South Atlantic, South Pacific and corresponding sector of the Southern Ocean. Usually, mixing models are used to evaluate the percentage contribution of potential prey items to the diet of consumers [46,66–71], and are increasingly being used to reconstruct dietary preferences of past populations from museum specimens using modern data on diet and isotopic signatures of major prey items [72–74]. As mammal bones demonstrate relatively slow metabolic turnover rates, isotopic composition of bone collagen would reflect the integration of dietary inputs over years [75,76]. If the studied consumer species is a specialist feeder, and prey objects show wide geographical distribution, then isotopic signatures of consumer might be relative to the primary feeding grounds.

Baleen whales often forage in productive areas, and various populations within the recognized subspecies rely on different areas with contrasting oceanographic characteristics and prey species which can be traced using stable isotope analysis [35,77]. For baleen whales, krill (Euphasiacea) is the main dietary source [78,79], and in the studied region taxonomic diversity and isotopic signatures of krill vary with latitude and among regions [48]. The southwest Atlantic sector of the Southern Ocean is considered as the region with highest stocks of Antarctic krill *Euphasia superba* [80–82], high productivity and associated krill abundances are also typical of the Subantarctic islands, e.g. South Georgia [3], and across both the western and eastern Patagonian shelf [83–85]. By using stable isotope data of krill and pelagic fishes from various latitudes and regions, a recent study reconstructed foraging patterns of Antarctic blue, fin and sei whales in the Southern Hemisphere [11,63]. Because for most of the specimens in our dataset, the geographical origin indicates the unloading location from the whaling vessel, which may be far from the actual catch site, and considering the high migratory abilities of whales, we assumed equal weightings (i.e. equal model default probability of diet contribution) for the MixSIAR models in this study.

Mixing models, considering equal weightings of the potential prey items, suggested potential feeding ground fidelity in some of the blue whale specimens in our dataset, i.e. the accordance between the NHMUK label indicating the geographical origin of specimen and the model prediction of feeding ground. Yet, for the most specimens model predicted feeding at various locations within our study region. Two recognized subspecies of blue whales are recognized in the Southern Hemisphere and northern Indian Ocean: Antarctic (or true) blue whales (*B. musculus intermedia*) and temperate-latitude pygmy blue whale *B. musculus brevicauda*, with prevalence of Antarctic blue whales to the south of 52°C [86,87]. The mixing model indicates high fidelity in at least one of the 1870s whales from the Strait of Magellan to Chilean sources, most likely taxonomically assigning it to the eastern South Pacific population of pygmy subspecies [60]. By contrast, the whales from South Georgia and the South Shetlands mostly have isotopic signatures of typical Antarctic blue whales based on their modelled dietary sources (figure 5). Nevertheless, most of the specimens demonstrate lack of clear fidelity to one location. These results corroborate the findings of a recent study based on mark-recovery models [88]. By using the Discovery Investigations and later mark data obtained between 1926 and 1963, the authors identify the high probability of blue whale movement between all sectors of the Southern Ocean, indicating no mark-recovery pairs with links between the Antarctic and any temperate regions [88]. Our results suggest that some blue whales could potentially undertake foraging migrations between Antarctic and Patagonian waters, as mixing models predicted partial contribution of temperate and Subantarctic sources in several Antarctic and Subantarctic specimens, and vice versa, Antarctic source contribution in specimens from the Strait of Magellan. These findings need further verification using datasets with larger sample size. Yet, these model predictions do not contradict results of previous studies indicating that some blue whale individuals may forage at multiple locations across the Southern Hemisphere and can migrate seasonally to lower latitudes from Antarctic waters [63,89]. Similarly, rare findings of Antarctic genotypes in lower latitudes [60] also support this hypothesis.

The Antarctic blue whales were hunted nearly to extinction during the twentieth century, and modern population estimates are considered to be less than 1% of the pre-whaling period [90]. Thus, the modern feeding patterns of blue whales in the Southern Hemisphere are likely to be not completely similar to the pre-whaling period [63,88]. Recent genetic studies indicate that some Antarctic blue whales demonstrate signatures of admixture with populations from temperate latitudes [60], providing a plausible basis for the interpretation of past connectivity between these populations or presence of long-distance migrants outside Antarctic waters.

The *δ*^15^N values from bulk stable isotope analysis cannot be compared directly among tissues derived from food webs in different areas [38,91], but the comparisons are possible through calculating TP based on *δ*^15^N values of lower trophic levels, or POM in aquatic ecosystems [40,44,63]. Our data indicate that none of the standard calculation approaches for converting bulk nitrogen isotope data to TP estimates can be used alone for the blue whale dataset consisting of specimens from various populations and from regions with highly contrasting productivity (figure 3). Of the three tested, the most conventional approach [38] provided the most realistic estimates of TP compared to those previously described for the baleen whales. The applied approach of using of POM values that correspond to the region of origin of the studied whale specimens, and the observed mismatches between the expected and observed TP values show, along with the Bayesian mixing models, the mismatch between the indicated origin of specimen (e.g. landing from the whaling vessel) and potential feeding grounds in whales. Currently, a more promising way of calculating the TP in whales and other higher marine consumers is based on data from compound-specific stable isotope analysis (CSIA) of amino acids [92]. In the last decade, stable isotope analysis of specific compounds within tissues, such as individual amino acids, has become increasingly common in archaeological, trophic and animal migration studies [93,94], and when possible, comparisons of bulk and CSIA are being implemented [95].

Nevertheless, the combined use of TP estimations and mixing models can help explain the origin of the variable results derived from museum specimens. In our dataset, the unexpectedly high *δ*^15^N (17.0‰) and correspondingly high TP value (4.5) were obtained for the specimen from the Strait of Magellan, for which the mixing models predicted fidelity to the Chilean pygmy blue whale population (figure 5C). While this *δ*^15^N value is very different from any of the Antarctic consumers, it is very close to values from fin whales from northern Chilean waters (17.9‰ in the winter), where nitrogen signatures of all elements of the pelagic food web are higher compared to subpolar and polar regions [96]. Increased *δ*^15^N values can be also typical of calves still suckling *δ*^15^N-enriched milk or in starving individuals [97]. Finally, environmental DNA studies of from the North Atlantic suggest that baleen whales including the blue whale, generally considered as highly specialized krill consumer, can forage for mesopelagic schooling fish ascending to the epipelagic zone at night [98]. Thus, further investigation and re-identification of this specimen is required for better understanding of the observed deviant isotopic signatures.

Our dataset includes four fin whale specimens collected between 1912 and 1951 in South Georgia, the South Shetlands and in the South Atlantic. These specimens also demonstrate pronounced variation in carbon and nitrogen isotopes, and while these results can be considered preliminary, they corroborate results from a recent baleen plate isotopic study concluding that the blue and fin whale in the Southern Ocean occupy a similar trophic level but demonstrate niche differentiation [63]. One potential explanation for the observed high TP for several fin whale individuals is similar to those for the outlier blue whale specimens, i.e. their feeding grounds could be far from the catch locations, or some individuals might undertake seasonal migrations to lower latitudes where their diet changes [11,63]. An additional and not mutually exclusive explanation might imply that the diet of fin whales in various parts of the distribution range include ichthyoplankton and small pelagic fish in addition to krill, and that in Patagonian waters, the zooplankton consumed by whales include larvae of squat lobsters *Munida gregaria*, all occupying higher a trophic level than krill [53,63,99].

Our data indicate changes in the diet of the South American fur seal from the Falkland Islands against a background of consistent regional *δ*^13^C values. Estimates of TP reveal a significant change in the trophic preferences of fur seals in the Falkland Islands from the nineteenth to the twentieth centuries. The diet of the South American fur seal includes both coastal and open-sea shelf prey items [100]. Currently, the dominant prey items of fur seals in the Falkland Islands , according to scat analysis, are Falkland herring *Sprattus fugensis*, and several other taxa including Patagonian squid *Doryteuthis gahi*, and several rock cod species *Patagonotothen* spp*.* [55]. While piscivorous fish species, e.g. *Merluccius hubbsi* are rarely found in the diet of fur seals [55,100], these were included among other potential sources in our mixing model (figure 5), primarily as an outgroup, and because recent studies based on satellite tracking data indicated long-distance foraging migrations in several colonies of the South American fur seal [54]. Calculation of TP and illustration of dietary preferences using the mixing models both indicate that South American fur seals could have had more selective feeding in the nineteenth century, and more opportunistic feeding in the twentieth century, possibly owing to a decline in previously preferred prey species. Seals from the 1870s could presumably be foraging for larger prey from higher trophic levels. As several fish taxa in Patagonian waters have experienced overfishing [101–103], our data could indicate that observed changes in the diet of the South American fur seal represent an adaptation to changes in the pelagic ecosystem.

The leopard seal is a species capable of foraging on taxa at various trophic levels, including other seal species, penguins, fishes and krill [104–106]. Huge variation in dietary preferences should affect the TP in different locations, seasons and even particular individuals. The highest TP in leopard seals was obtained for the Ross Sea specimens (4.5 ± 0.34); and lowest TP value was obtained for two studied specimens from the Falkland Islands (2.2 ± 0.36). No significant temporal differences in TP were found in leopard seals because differences within each temporal subset are higher than that between the subsets (figure 4). According to our data, the TP of leopard seals at the Falkland Islands is nearly twice as lower compared to the South American fur seal. Based on stable isotope analysis and scat data, a recent study of leopard seals at the Antarctic Peninsula show that the diet of this species largely consist of krill and notothen fish during the seasons when mesopredator prey (e.g. penguins) is less available [105]. Despite the small sample size (*n* = 1 for Antarctic peninsula and *n* = 2 for Falkland Islands; figure 4), our data might suggest that the diet of studied leopard seal specimens from the Falkland Islands largely consisted of krill and low trophic level fish. Yet, these data should be considered preliminary and verified using larger sample sizes.

## 5. Conclusions

Bulk stable isotope analyses of museum specimens in this study provide a baseline record of Patagonian, Subantarctic and Antarctic marine ecosystems before and during the period of active exploration and exploitation of marine resources in the region at the turn of the twentieth century. Here, we show how this dataset can be used to address questions about the influence of industrial whaling, seal hunting and the development of fishing on the foraging of whales and seals. This study discusses the possible constraints for using this dataset in the context of conventional ecological analyses aimed at understanding the long-term changes in Antarctic marine ecosystem using several keystone species. Our results suggest that prior to and during the onset of industrial whaling in Antarctica blue whales used a wide variety of foraging areas with some individuals potentially undertaking foraging migrations between the Antarctic and lower latitudes. Also, some South Pacific blue whales could have undertaken foraging migrations into Subantarctic and Antarctic waters during the late nineteenth century, similar to modern populations. The South American fur seal population in the Falkland Islands could have foraged for a prey from higher trophic levels before the onset of industrial fishing in the region, and the leopard seal data demonstrate a high degree of adaptation to the local availability of prey objects. These hypotheses, based on the conventional bulk stable isotope analysis, should be further verified on the basis of larger sample sizes for each species and compared with modern data from the Antarctic and other regions within the species ranges. Also, application of more advanced methods such as compound-specific isotope analysis of amino acids can be helpful for distinguishing proxies of primary producers for internal calibration of TP. Improved accuracy in estimating the TPs of past Southern Hemisphere common whale and seal populations would provide critical information regarding the long-term fluctuations or consistency in their foraging strategies, providing greater resolution to the analysis of food webs and implications for the monitoring and conservation of these species.

## Data Availability

All the raw data for this paper are presented in the electronic supplementary material [107].
